# A Novel Bioimplant Comprising Ad-BMP9-Transfected BMSCs and GelMA Microspheres Produced from Microfluidic Devices for Bone Tissue Engineering

**DOI:** 10.1155/2023/2981936

**Published:** 2023-06-19

**Authors:** Li Nie, Wei Liu, Jiajun Chen, Siqi Zhou, Chang Liu, Wenhui Li, Zhiyue Ran, Yaxian Liu, Jing Hu, Yuxin Zhang, Liwen Zheng, Ping Ji, Hongmei Zhang

**Affiliations:** ^1^Stomatological Hospital of Chongqing Medical University, Chongqing 401147, China; ^2^Chongqing Key Laboratory of Oral Diseases and Biomedical Sciences, Chongqing Municipal Key, Chongqing 401147, China; ^3^Laboratory of Oral Biomedical Engineering of Higher Education, Chongqing 401147, China

## Abstract

Oral and maxillofacial bone defect repair in patients remains challenging in clinical treatment due to the different morphologies of bone defects. An injectable hydrogel of microspheres with sustained bone morphogenetic protein 9 (BMP9) expression for oral and maxillofacial bone defect repair has been developed. This study is bioinspired by the substantial osteogenesis property of recombinant adenoviruses expressing bone morphogenetic protein 9 (Ad-BMP9) and minimally invasive treatment by injection. A novel scaffold encompassing bone mesenchymal stem cells (BMSCs) transfected with Ad-BMP9 was produced and cocultured on a superficial surface of monodisperse photocrosslinked methacrylate gelatin hydrogel microspheres (GelMA/MS, produced with microfluidic technology). The biological tests including live/dead cell staining, phalloidin staining, cell counting kit-8 (CCK-8) assay, alkaline phosphatase (ALP) activity and staining, alizarin red S staining, and quantitative real-time polymerase chain reaction (RT-qPCR), revealed that the hydrogel microspheres exhibited good biocompatibility and remarkably promoted the osteogenic differentiation of BMSCs *in vitro.* In addition, a small needle was injected the innovative scaffold beneath the nude mice's skin. The micro-CT and histological staining assay results demonstrated that the new implant, with high blood vessel formation markers (CD31-positive cells) expression over four and eight weeks, achieved significant vascularized bone-like tissue formation. Consequently, the injectable hydrogel microspheres, cocultured with BMSC transfected with Ad-BMP9, enhanced vascularized bone regeneration, therefore representing a facile and promising technique for the minimally invasive treatment of oral and maxillofacial bone defects.

## 1. Introduction

Bone loss is common in patients undergoing oral and maxillofacial surgery. The primary causes of bone tissue loss are trauma, tumours, periodontitis, and periapical inflammation. The conventional treatment of maxillofacial bone defects involves implanting bone substitutes by surgery. However, the surgical operation induces considerable trauma, resulting in severe pain and an extended recovery time. Therefore, scope exists for a highly effective and minimally invasive treatment, such as injections. Consequently, developing an innovative nonsurgical invasive technique to achieve bone regeneration is exceedingly desirable.

In biomedical engineering, tissue engineering as a potential alternative to tissue transplantation has attracted considerable attention and developed rapidly [1, 2]. At the same time, there are many challenges in tissue engineering, such as invalid tissue regeneration, challenges of using it clinically, and a lack of biomaterials with ideal mechanical, chemical, and biological properties [3, 4]. Several hydrogels developed for bone tissue engineering, such as alginate [5], chitosan [6, 7], fibrin [8, 9], collagen [10], and hydrogel composites (nanoparticles, nanofilms, and nanocomposites) [11–15], have attracted much attention in tissue engineering. Most hydrogels have the disadvantages of poor mechanical properties and limited cell adhesion. In recent years, injectable hydrogels have been developed with excellent biocompatibility, strong cell adhesion, and dynamic physicochemical properties in tissue engineering [16–18]. Methacryloyl functionalized gelatin (GelMA) is a famous biomaterial that is a gelatin derivative [13]. Gelatin is derived from collagen and resembles the native extracellular matrix (ECM) due to its remarkable cell adhesion and matrix-metalloproteinase- (MMP-) responsive amino acid motifs [13]. These features make gelatin-based scaffolds great candidates for biomedical applications, since collagen is the most abundant protein in the skins, bones, cartilage, and connective tissue [13, 19]. Under UV or visible light irradiation, the GelMA photocrosslinks into bulk hydrogels with high water content and porous structures at the nanometre level. However, an inevitably high and uneven injection force might cause damage to the healthy tissue and discomfort to the patient during the injection process, owing to the uneven shape and large hydrogel size [20]. It can be challenging to administer bulk GelMA hydrogels via injection for minimally invasive applications [21, 22]. Microfluidic technology is used to engineer GelMA/MS with significantly enhanced structural stability and high injectability because of the spherical shape and relatively uniform size [23] in a highly efficient and controllable manner [24–26]. Compared with conventionally bulk GelMA, (I) GelMA/MS retains the fundamental properties of bulk hydrogels and can be applied for minimally invasive applications by small needle injection, making them suitable for complex, irregular, and minute bone defects, (II) GelMA/MS still provides a 3D microenvironment similar to those of the natural extracellular matrix (ECM); GelMA/MS often exhibit enhanced cell viability and functions due to better oxygen and nutrition conditions [27], and (III) using GelMA/MS for delivering cells is a reliable and protective engineering strategy for avoiding the low survival rate, cell damage, faster cell death, and rapid dislocation caused by direct cell injection [28]. Previous studies, however, have mainly focused on the property as a vehicle that aims to achieve sustained osteogenesis protein expression for vascularized bone regeneration.

Bone morphogenetic proteins (BMPs) are members of the TGF-*β* superfamily, and BMP9 is one of the most osteogenic BMPs that promote the osteoblastic differentiation of mesenchymal stem cells (MSC) *in vitro* and *in vivo* [29]. Wang reported that BMP9 facilitates fracture healing in osteoporotic conditions. Wang [30] revealed that BMP9 and P-15 peptide hydrogels assisted in repairing rabbit femoral defects using 3D-printed polylactic/poly glycolic acid (PLGA) scaffolds. Osteogenesis and angiogenesis are coupled during bone development, which is well recognized [31, 32]. Blood vessels carry oxygen and nutrients to all cells and remove waste products from the tissue. Angiogenesis is therefore a prerequisite for osteogenesis [33, 34]. A previous study showed that sinusoid capillary formation was observed early in BMP9-induced bone formation [35]. BMP9-induced bone formation is coupled with angiogenesis, which promotes both osteogenesis and angiogenesis. Therefore, BMP9 can be an excellent bone regeneration factor in bone tissue engineering.

Currently, the delivery of drugs and cytokines through nanocomplexes is more widely used in tissue engineering, such as nanoparticles and nanofilms [6, 12, 13, 36]. Direct delivery of drugs, proteins, or cytokines may cause local protein concentrations that are too high. For example, exogenous recombinant bone morphogenetic proteins (rBMPs) directly act on cells and will lead to the rapid and irregular ectopic formation of bone nodules, causing ischemia in the central area and necrosis, resulting in aseptic inflammation, body and function problems, and directly affecting bone formation. Even severe cases can lead to the production of tumors. The development of tumors cannot be controlled, leading to severe clinical problems.

Importantly, viral vectors for gene delivery have become the most popular vectors for high efficiency [37]. Delivery of the gene via adenoviral vectors can be better controlled and more efficient than delivery of the protein [37]. The osteogenic activity induced by recombinant human bone morphogenetic protein 9 (rhBMP9) was significantly weaker than Ad-BMP9 [38]. For example, Li [39] highlighted that Ad-BMP9 could enhance bone regeneration in alveolar bone defects using hydroxylapatite as scaffolds. However, no studies have explored how combining Ad-BMP9-transfected BMSCs and GelMA/MS scaffolds can effectively promote bone formation *in vivo* by further promoting blood vessel formation via injection.

As depicted in the graphical abstract, the current study proposes an innovative composite biomaterial by synthesizing injectable hydrogel microspheres with BMSCs transfected with Ad-BMP9 (BMSCs-GelMA/MS-BMP9) for bone regeneration. To begin with, GelMA/MS were produced from microfluidic devices and were cocultured with BMSCs transfected with Ad-BMP9. Subsequently, a small needle injection injected the biomaterial beneath the nude mice's skin. The novel composite biomaterial remarkably promoted the osteogenic differentiation of BMSCs *in vitro*. Furthermore, with enhanced vessel formation over four and eight weeks, the compound achieved significant bone beneath the skin of nude mice by micro-CT and histological staining assay. These data infer that the BMSCs-GelMA/MS-BMP9 developed herein may represent a facile and promising approach for vascularized bone regeneration.

## 2. Materials and Methods

### 2.1. Synthesis of GelMA

The GelMA was synthesised using a similar method reported previously [40–42]. Briefly, type A porcine skin gelatin (G8060, Solarbio, China) was fully dissolved in phosphate buffer saline (PBS, pH = 7.4) at 60°C and a concentration of 10% (w/v). A specific amount of 10% (v/v) methacrylic anhydride (MA, 64100, Sigma-Aldrich, USA) was added dropwise to the solution. The components were stirred at 50°C to react for 2 hours. About 2× dilutions with additional PBS were performed to stop the reaction. The resulting solution was dialysed against distilled water at 40°C for seven days using a dialysis membrane (8–14 kDa, YA1073, Solarbio, China). The GelMA solution was filtered using a 0.22 *μ*m filter membrane and kept at −20°C for 24 h, then lyophilized for seven days to obtain a white porous foam solid, which was subsequently stored at −20°C.

### 2.2. Microfluidic Fabrication of GelMA Microspheres

GelMA/MS were prepared using two microfluidic devices (LSP04-1A, Longer, USA). The GelMA solution and mineral oil (mineral oil: span 80 = 9 : 1, 8020-83-5,1838-43-8, RHAWN, China) were produced by the microfluidic system as the water and oil phases, respectively. The main principle is to regulate the velocity of the oil and GelMA phases and create microdroplets by shearing the GelMA phase relative to the fast-flowing oil. Finally, the liquid droplets were transformed into solid microspheres by UV light (EFL-LS-1601, EFL, China) and washed with PBS thrice. The diameter of microspheres was measured to document the size distribution.

### 2.3. Characterization of GelMA/MS

#### 2.3.1. The Diameter of GelMA/MS and Swelling Analysis

The particle size and morphology of GelMA/MS after freeze-drying and swelling for 10 min and 24 h were analysed using ImageJ software. The freeze-dried microspheres were weighed to measure the swelling of hydrogels microspheres, and the weight was recorded as *M*_*0*_. Then, all the samples were immersed in PBS at 37°C for 10 min and 24 h. The excess water was removed, and the swollen weight (*W*_*t*_) was recorded. The freeze-dried GelMA, with the same weight was taken as the control. Also, 6 samples were prepared in each group. The mass swelling ratio was calculated using the following equation:

(1) $$\begin{array}{c} R=\frac{\left(W_{t}\;–\;W_{0}\right)}{W_{0}\times 100\%}\text{.} \end{array}$$

#### 2.3.2. Degradation *In Vitro*

In total, 2 *μ*M collagenase type II was used to investigate the degradation of GelMA/MS *in vitro.* The GelMA/MS samples with determined weight (*M*_*0*_) were placed in collagenase type II solution on a shaker at 37°C. The samples were centrifuged at distinct time points and weighted again (*M*_*t*_) after discarding the liquid. In addition, 7 samples were prepared in each group. The mass degradation rate was calculated using the following equation:

(2) $$\begin{array}{c} Degrada\;tion\;rate=\frac{\left(M_{0}-M_{t}\right)}{M_{0}\times 100\%}\text{.} \end{array}$$

#### 2.3.3. Equipment Analysis

The GelMA/MS were freeze-dried for three days. They were dissolved in D_2_O to yield a 5 mg/ml concentration, and the data were processed and analyzed in MestreNova NMR software. DSA30 was used to measure water contact angle. To confirm the crystal phase, samples were freeze-dried for three days for SEM, FTIR, and XRD analysis. SEM images were analyzed using ImageJ software to calculate porosity.

#### 2.3.4. Thermogravimetric Analysis of Hydrogel (TGA)

GelMA and composite hydrogels were used for TGA. Hydrogel was freeze-dried after freezing at −80°C. The thermal behaviour of the samples was carried out on an SDTQ600 unit, with a 10°C/min heating lamp, from 35 to 800°C.

#### 2.3.5. Zeta Protein

The freeze-dried GelMA/MS and GelMA were immersed in PBS at 37°C for 10 min and Zen3600 was used to measure zeta potential.

### 2.4. Cell Culture

Primary BMSCs were obtained from the femur and tibia of Sprague–Dawley rats (four weeks old) as previously described [43]. The cells were cultured in a complete medium (DEME/F12, 10% fetal bovine serum, and 1% streptomycin/penicillin). All animals were purchased from the Experimental Animal Centre of Chongqing Medical University (License No: SYXK(Yu)2019-0004). All procedures in the present study were in accordance with the NIH guidelines on the ethical use of animals. The experiments in the study were approved by the Ethics Committee of Chongqing Medical University (CQLA-2021-0949). Cells (passage 3) were resuspended and recultured for further investigations. The method of Ad-BMP9 transfecting BMSCs was performed as previously described [39]. 80% confluence cells (passage 3) were digested by 0.25% trypsin and resuspended and recultured in a humidified environment of 5% CO2 for 3 hours. Transfection was then performed with Ad-BMP9 at an appropriate multiplicity of infection (MOI, transfection efficiency about 70%), followed by refreshing the medium in 4 hours. Ad-GFP was used as a negative control. Polybrene (1 *μ*M/mL, H8761, Solaibio, China) was added to enhance the transfection efficiency [44, 45]. The cells transfected with Ad-BMP9 or Ad-GFP were observed under a fluorescent microscope after 24 and 48 hours (SteReoDiscovery V20 Carl Zeiss, Germany). These cells were digested and centrifuged to cell suspension for the subsequent experiment. Both Ad-BMP9 and Ad-GFP were provided by Dr. TC He (Professor, University of Chicago Medical Centre, USA).

### 2.5. Biocompatibility of GelMA/MS

Aliquots of 100 mg microspheres per well (24-well plates) were immersed in the complete medium for 24h. The medium was then discarded. BMSCs were cocultured with GelMA/MS in 24-well plates at a density of 2 × 10^4^ cells/well. Also, 3 samples were prepared in each group. Live/dead cell staining was performed on days 1, 3, 5, and 7 using the live/dead cell staining kit (CA163, Solaibio, China). Phalloidin staining was performed on days 3, 5, and 7 using the phalloidin staining kit (C2203S, Beyotime, China). The staining images were taken through a fluorescence microscope. BMSCs were mixed with 100 *μ*L of the microsphere solution and cocultured in 96-well plates at a density of 2 × 10^3^ cells/well. CCK-8 (C0037, Beyotime, China) was tested on days 1, 3, 5, and 7, and the solution's optical density value (OD) at the wavelength of 450 nm was measured.

### 2.6. Osteogenic Activity of BMSCs-GelMA/MS-BMP9

BMSCs were mixed with 1 mL of the microsphere solution and cocultured in 24-well plates at a density of 2 × 10^4^ cells/well. Also, 3 samples were prepared in each group. ALP activity was detected using a chemiluminescence assay (P0321, Beyotime, China) on days 1, 3, 5, and 7 and ALP staining (P3206, Beyotime, China) on day 7. The staining results were recorded using an inverted microscope and camera. After 21 days, the cell-laden GelMA/MS were stained with alizarin red S dye (G8550, Solarbio, China), and an inverted microscope and camera recorded the results. After dissolving the mineralised ECM with 10% acetic acid, the OD values (405 nm) were measured. The total mRNA was extracted (R0026, Beyotime, China) on days 1, 3, 5, and 7, and cDNA was prepared (RR036A, TaKaRa, Japan). Real-time qPCR was used to quantify the osteogenic gene expression (alkaline phosphatase, *ALP*, runt-related transcription factor 2, *RunX2*, osteopontin, *OPN*) using the SYBR® Green Assay kit (RR820A, Takara, Japan). All samples were run in triplicate and normalised by glyceraldehyde-3-phosphate dehydrogenase (GAPDH) expression. The primers were synthesised by Genscript (Tsingke, China). The detailed primer information is presented as follows:

 
*ALP*, F:5′-GATGGTATGGGCGTCTCCAC-3′
  R:5′-CGTTGGTGTTGTACGTCTTGG-3′
 
*OPN*, F:5′-CAGTCGATGTCCCTGACGG-3′
  R:5′-GTGGCATCGGGATACTGTTCAT-3′
 
*RunX2*, F:5′-TGGCCGGGAATGATGAGAAC-3′
  R:5′-TGAAACTCTTGCCTCGTCCG-3′

### 2.7. Osteogenesis *in Vivo*

GelMA/MS, BMSCs-BMP9, BMSCs-GelMA/MS-GFP, and BMSCs-GelMA/MS-BMP9 implants were prepared for animal experiments to evaluate the osteogenic potential of Ad-BMP9 transfected with BMSCs and GelMA/MS *in vivo*. An ectopic bone formation animal model was selected for this study because this model would investigate whether the novel compound of Ad-BMP9-transfected BMSCs and GelMA/MS could support bone formation in such an environment without any stimulation. First, BMSCs transfected with Ad-BMP9 were cocultured with GelMA/MS for three days. Second, ectopic implantation was injected beneath the skin of nude mice. Then, 6 samples were prepared in each group. After 4 and 8 weeks, the implants were extracted and fixed in 4% paraformaldehyde (pH: 7.4) for 48 hours at room temperature before the micro-CT analysis. At least 20 random high-power fields of cross-sections per group were evaluated by a picture-analysis system (the Image-Pro Plus software) to calculate the percentage of new bone formation over the total defect for each group. The micro-CT settings were adjusted as follows: pixel matrix = 2048 × 2048; slice thickness = 10 *µ*m; FOV/diameter = 21.5 mm; energy/intensity = 70 KVp, 114 *µ*A, 8 W. The 3D images were reconstructed using *µ*CT-6.1 software (Scanco Vival CT 40, Switzerland). Briefly, the threshold (205–550, representing the grey value of new mineralisation tissue) of the grey value was selected. Another grey value threshold (<205) was regarded as GelMA/MS or soft tissue. The 3D reconstruction of each group (*n* = 6) and the amount of new bone tissue in the sample were also calculated. Hematoxylin and eosin (H&E) staining and Masson staining were used to evaluate the new bone formation. Anti-rat CD31 (GB11063-2, Servicebio, China) was used for immunohistochemical staining. ImageJ software was used to calculate bone volume fraction (BV/TV) and CD 31 positive cells.

### 2.8. Statistical Analysis

All the experiments were repeated thrice independently. The data were expressed as mean ± standard deviation (SD). Statistical significance was determined using a one-way analysis of variance followed by Tukey's post-test using GraphPad Prism software (GraphPad 9). A *p* value less than 0.05 was considered statistically significant.

## 3. Results

### 3.1. Microfluidic Fabrication of GelMA/MS

**Figure 1:**
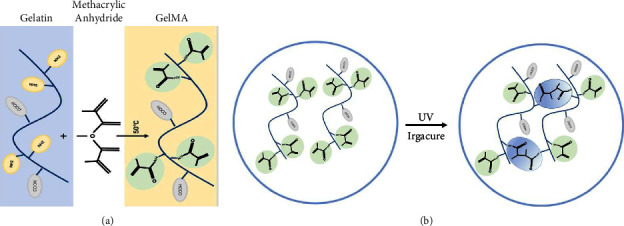
GelMA synthesis. The chemically modified gelatin contains methacrylate and methacrylamide groups which enable it to be photocrosslinked under UV light by the assistance of a photoinitiator.

**Figure 2:**
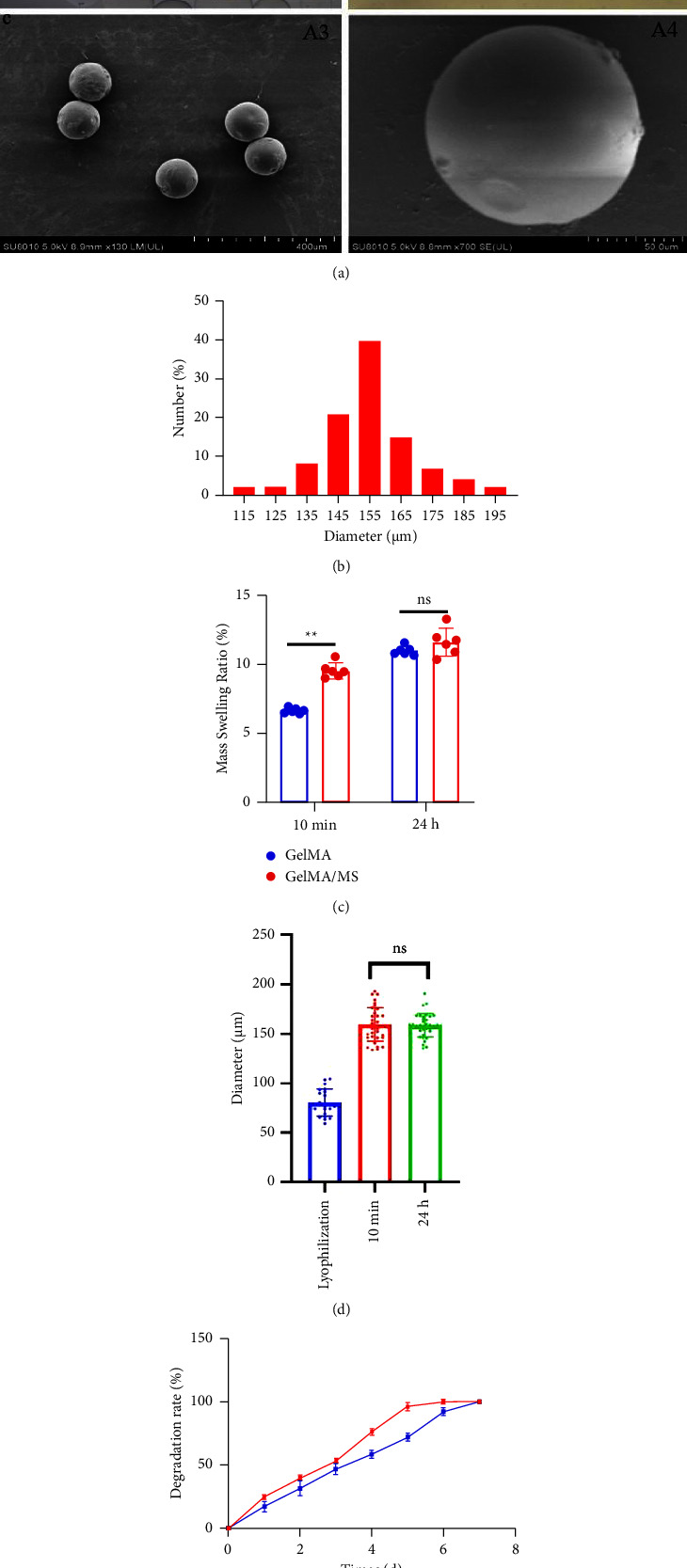
Physical characterization of GelMA/MS. (a) Representative images of GelMA/MS: dispersed microspheres (A1), mono-micro-sphere (A2) and SEM images of GelMA/MS (A3 and A4). (b) The particle size distribution of GelMA/MS. (c) The mass swelling ratio of GelMA and GelMA/MS (*n* = 6, ^*∗∗*^*p*  <  0.01). (d) The diameter of GelMA/MS before and after swelling (ns indicates *p* > 0.05). (e) Degradation analysis of GelMA/MS incubated with the II collagenase, GelMA and GelMA/MS biodegradation profiles.

GelMA/MS were produced using a coaxial needle and two microfluidic devices. The chemically modified gelatin contains methacrylate and methacrylamide groups, enabling it to be photocrosslinked under UV light with the assistance of a photoinitiator (Figure 1). Moreover, microspheres were monodispersed with good structural integrity, and the lyophilised GelMA/MS were observed under SEM (Figure 2(a)). In addition, the flow rates of both the continuous phase (*V*_*c*_) and dispersed phase (*V*_*d*_) are critical for regulating the sizes of emulsion droples [46]. GelMA/MS with an average diameter of 154.73 ± 14.65 *μ*m (range: 110*–*200 *μ*m) was obtained by adjusting the flow rate of two phases (*V*_*d*_ = 20 *μ*L/min, *V*_*c*_ = 700 *μ*L/min) (Figure 2(b)).

### 3.2. Characterization of GelMA

#### 3.2.1. Thermal Properties and Porosity

The thermal properties of the hydrogel were investigated using a thermal analyzer. The TGA curve shows that the hydrogel began to lose integrity between 50°C and 600°C, and the mass decreased (Figures S1A and S1B). The GelMA hydrogel porosity was assessed, establishing that GelMA has a large pore structure, with porosities of 35.95% ± 5.42% and 33.47% ± 6.24% (Figure S1C, *p* > 0.05).

#### 3.2.2. Zeta Potential

The Zeta potential was measured to verify the realization of scaffold tunable surface charge in the polymer. The surface of GelMA in double distilled water had a negative potential value of −2.14 mV and −2.5 mV (Figure S1D, *p* > 0.05), consistent with previous findings [47].

#### 3.2.3. The Hydrophilicity of GelMA/MS

The water contact angle is vital for determining hydrophobicity, an essential factor in biomedical applications [48]. As shown in Figure S2A, the hydrophilicity of GelMA/MS (water contact angle of about 39.16° ± 7.6°) was significantly improved compared with large hydrogels (water contact angle is approximately 67.6° ± 5.41°).

#### 3.2.4. Chemical Properties

To characterize the structure of GelMA/MS, diffraction patterns of GelMA/MS and GelMA were analyzed. The XRD results are shown in Supplementary Materials (Figure S2B), with no significant difference between the two groups. The strong peak at 1629 cm^−1^ is related to C=O stretching vibration, while the peak at 1382 cm^−1^ is related to C–N stretching plus N–H bending. The peak at 1053 cm^−1^ is associated with C–O stretching vibration (Figure S2C). The GelMA hydrogels contained abundant amino and amide groups from the FTIR spectra, confirming their high hydrophilicity. The XPS analysis experiment detected changes in the elemental composition of GelMA/MS and GelMA. The experimental results show that both GelMA/MS and GelMA elements contain elemental C, N, and O (Figure S2D).

### 3.3. Hydrogel Swelling and Degradation

The swelling characteristics significantly affect hydrogels' physical and mechanical properties, such as surface properties, surface mobility, and solute diffusion [49]. The mass swelling ratio of GelMA/MS reached 9.59% ± 0.71% and is higher than the GelMA group (*p*  <  0.01) at 10 minutes (Figure 2(c)). However, no significant difference is present between the two groups at 24 hours. The average diameter of GelMA/MS is 80.40 ± 13.80 *μ*m after being lyophilised, 159.14 ± 17.04 *μ*m, and 159.44 ± 10.71 *μ*m after being soaked in PBS for 10 minutes and 24 hours, respectively (Figure 2(d)). The results indicate that GelMA/MS could absorb higher amounts of water and cause a higher degree of swelling at the beginning of the 10-minute immersion. To investigate the degradation rate of GelMA/MS, the same mass weight of GelMA was used as the control group, and collagenase type II was used to determine the degradation profile of hydrogel scaffolds [50]. GelMA and GelMA/MS samples are degraded entirely in 6 and 7 days, respectively (Figure 2(e)). The degradation rate for both samples is faster in the later stages than in the early stages, with the GelMA group taking longer to degrade than the GelMA/MS group.

### 3.4. Cell Culture

**Figure 3:**
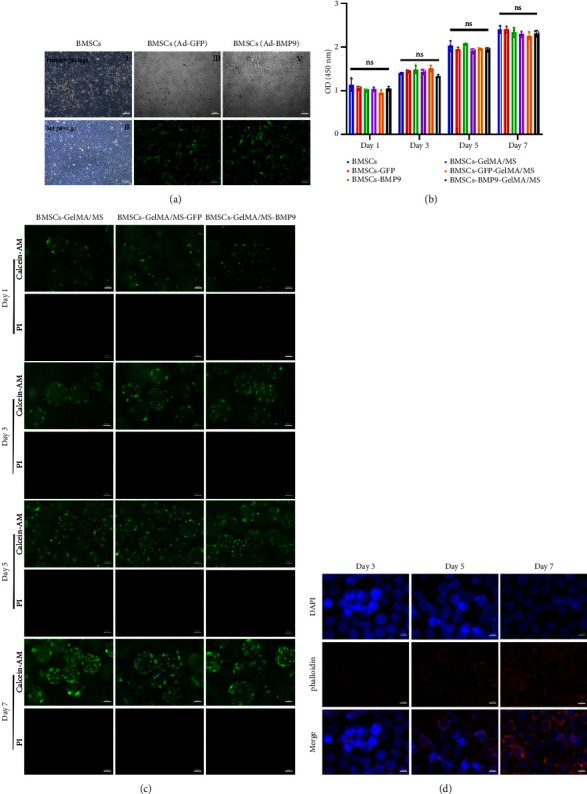
The biocompatibility of GelMA/MS. (a) The primary BMSCs and BMSCs transfected by Ad-BMP9. The primary and 3rd passage BMSCs showed in Figure 3(a) I and II. The shape and fluorescence of BMSCs transfected with Ad-BMP9 or Ad-GFP were showed in Figure 3(a) III, IV, V, and VI. (b) The cell counting kit-8 assay (*n* = 3, ns indicates *p*  > 0.05). (c) The live/dead staining images. The live cells are in green, and the dead cells are in red. (d) The fluorescence image of BMSCs seeded on monodispersed GelMA/MS at days 3, 5, and 7. Nuclei and GelMA/MS are blue (DAPI) and cytoskeleton is red (phalloidin).

The bone marrow of the tibia and femur rats was cultured as described in other research work [43, 51, 52]. The primary BMSCs are polygonal and fusiform (Figure 3(a) II). After the third passage, BMSCs appeared in fibroblast-like fusiform under the inverted microscope (Figure 3(a) II). The green fluorescence was regarded as a mark of Ad-BMP9-transfected BMSCs successfully. After 24 hours in culture, the fluorescence expression increased, and the transfection efficiency was about 70%–80% without changes in cell shape (Figure 3(a) III–VI).

### 3.5. The Biocompatibility of GelMA/MS

It is essential to investigate the biocompatibility of GelMA/MS by live/dead cell staining, phalloidin staining, and CCK-8 assay. Each group's OD values (Figure 3(b)) gradually increased in the CCK-8 assay on days 1, 3, 5, and 7. However, no significant difference was present among the groups (*p*  >  0.05). The viability of the compound was examined by live/dead staining assay (Figure 3(c)). Clearly, the live cells (green staining) exhibiting favourable viability and dead cells (red staining) were hardly observed in all groups at every time point. Notably, the cell density gradually increased over time. The phalloidin staining revealed that BMSCs could spread and proliferate well on the surface of the GelMA/MS over time (Figure 3(d)). On day seven, the cell ECM (red staining) and nucleus (blue staining) completely enveloped the GelMA/MS. Interestingly, GelMA/MS produced autofluorescence as blue. All these findings validate that the transfection of Ad-BMP9 and coculture with GelMA/MS had no apparent effect on cell adhesion, growth, and proliferation. The GelMA/MS exhibited good biocompatibility and no cytotoxicity.

### 3.6. Osteogenic Evaluation *In Vitro*

**Figure 4:**
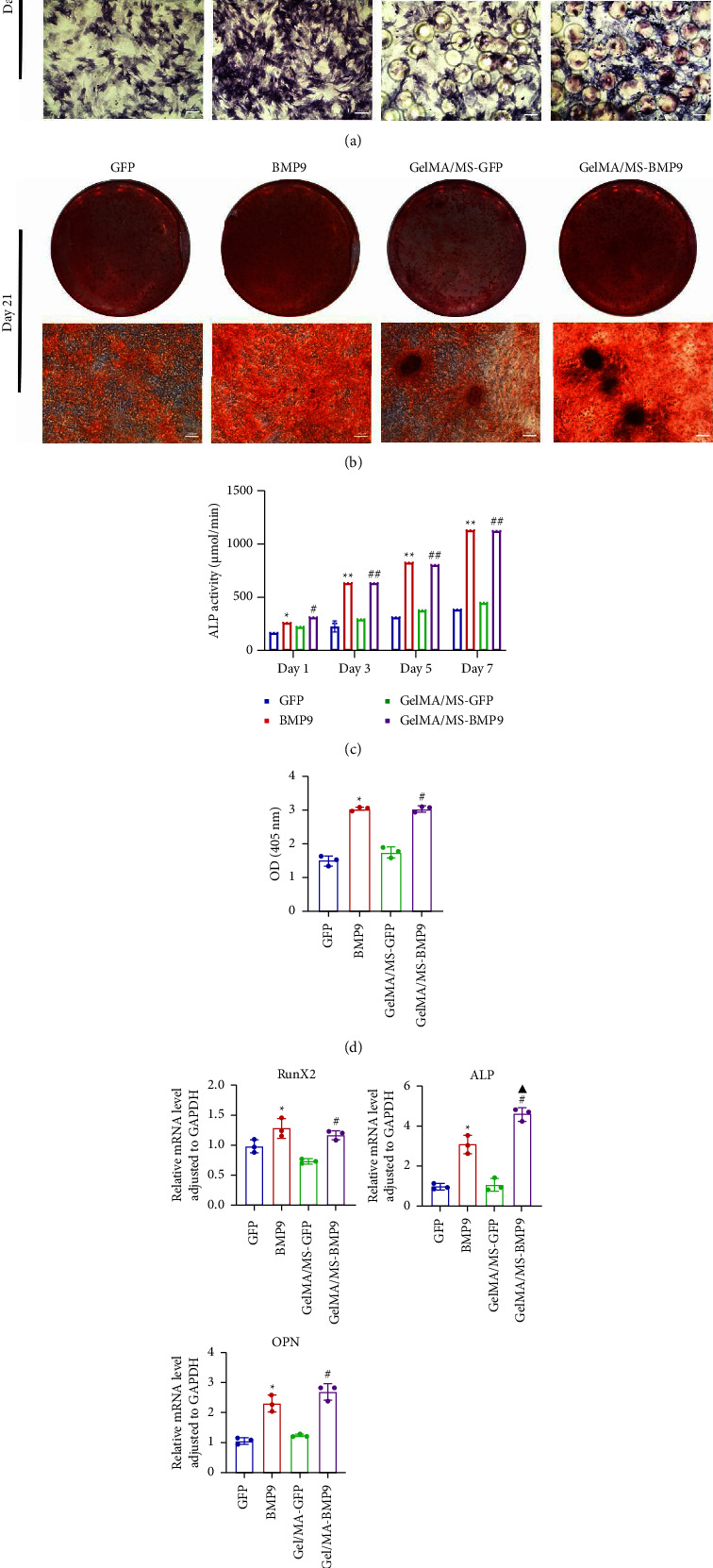
Osteogenesis *in vitro*. (a) Overall photograph and optical microscopic image of ALP staining on day 7. (b) Overall photograph and optical microscopic image of alizarin red S staining on day 21. (c) The activity of ALP (*n* = 3, ^*∗*^ and # indicate *p*  <  0.05, and ^*∗∗*^ and ^##^ indicate *p*  <  0.01 in comparison with GFP group and GelMA/MS-GSP group, respectively). (d) The quantification of calcium nodule (*n* = 3, ^*∗*^ and ^#^ indicate *p*  <  0.05 in comparison with GFP group and GelMA/MS-GSP group, respectively). (e) Real-time qPCR quantitative analysis of osteogenic genes including runt-related transcription factor 2 (*Run X2* on day 1), alkaline phosphatase (*ALP* on day 1), Osteopenia (*OPN* on day) (*n* = 3, ^*∗*^, ^▲^ and ^#^ indicate *p*  <  0.05 in comparison with GFP group, Gel-MA/MS-GFP group, and BMP9 group, respectively).

ALP activity and staining were investigated to evaluate the osteogenesis ability of Ad-BMP9 transfected BMSCs cocultured with GelMA/MS at different time intervals. ALP staining (the more intense colour, the higher the ALP activity) demonstrated ALP expression in the BMP9-treated groups (BMP9 group and GelMA/MS-BMP9 group) significantly increased compared with the GFP-treated group (GFP group and GelMA/MS-GFP group) on day seven (Figure 4(a)). The ALP activity analysis showed similar results (Figure 4(c)). Alizarin red staining (the deeper the colour, the more extracellular calcium nodules deposited) at 21 days was conducted to assess the late mineralisation levels of the novel composite biomaterial (Figure 4(b)). More calcium salt and nodules were stained red in the BMP9-treated group than in the GFP-treated group. This outcome confirmed that BMP9 promotes calcium salt deposition during the late osteogenesis of BMSCs cocultured with GelMA/MS. The result was further confirmed with quantitative analyses (Figure 4(d)). Furthermore, the expression levels of osteogenic genes (*RunX2, OPN,* and *ALP*) were detected using RT-qPCR (Figures 4(e) and S3). The expression levels of *RunX2*, *OPN,* and *ALP* in the BMP9-treated group increased compared with the GFP-treated group, suggesting that BMP9 enhances the osteogenic differentiation of BMSCs. In detail, the expression of Runx2, an early osteogenic regulator [53–56], is higher in the GelMA/MS-BMP9 group than in the BMP9 group on day 5(Figure S3). Compared with BMP9 group, there is a significant difference in the gene expression of *ALP* in GelMA/MS-BMP9 group on days 5 and 7, while it is 1.49-fold times and 1.56-fold times on days 1 and 3(Figure S3). These results reveal that GelMA/MS enhances the osteogenic effects of BMP9 on BMSCs. All these findings suggest that BMP9 promotes the osteogenic differentiation of BMSC, and GelMA/MS could improve the outcome *in vitro*.

### 3.7. Osteogenesis *In Vivo*

**Figure 5:**
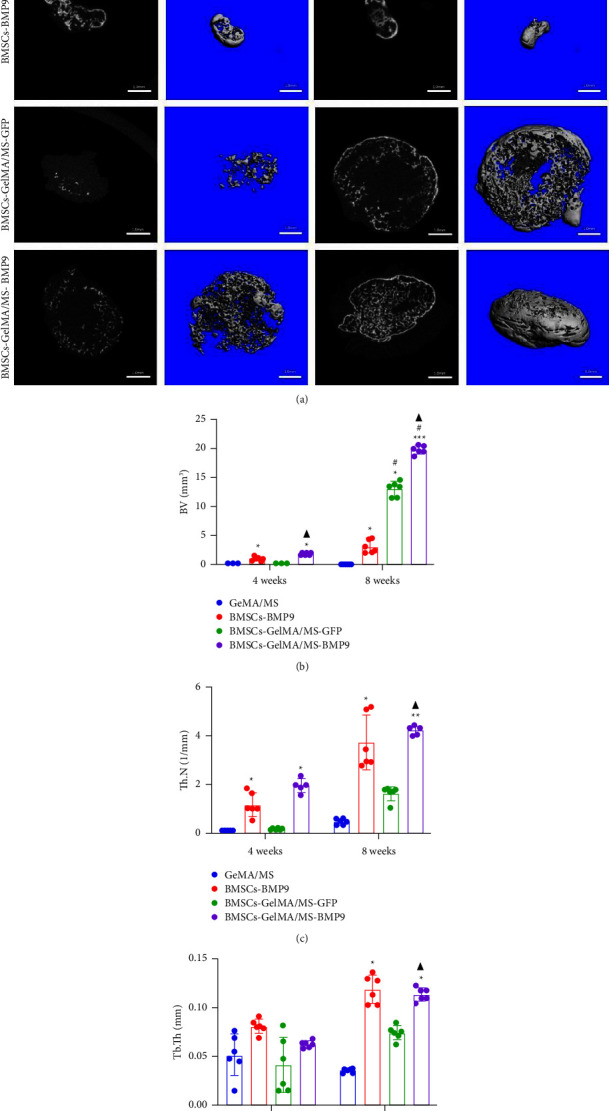
The micro-CT 3D-reconstructed images and quantitative analysis of new bone. (a) Micro-CT 3D-reconstructed images of new bone. (b–d) Quantitative analysis of bone volume (BV), number of trabeculae (Th.N), trabeculae thickness (Tb.Th) in different groups at weeks 4 and 8 (bars = 100 *µ*m in BMSCs-BMP9 group and 1 mm in other groups), (*n* = 6, ^*∗*^, ^#^, and ^▲^ indicate *p*  <  0.05 in comparison with the GelMA/MS group, the BMSCs-BMP9 group, and the BMSCs-GelMA/MS-GFP group, ^*∗∗*^ and ^*∗∗∗*^ indicate *p*  <  0.01 and *p*  <  0.001 in comparison with GelMA/MS group, respectively).

GelMA/MS, BMSCs-BMP9, BMSCs-GelMA/MS-GFP, and BMSCs-GelMA/MS-BMP9 implants were created and delivered beneath the skin of mice with immunodeficiency by injection, using a 26G needle syringe, to evaluate the osteogenic potential of new composite biomaterial *in vivo*.  Figure S4 shows that the volume of implants in the BMP9 group was the smallest within the samples, indicating that GelMA/MS plays a supportive role in the new bone tissue. The micro-CT 3D-reconstructed images and quantitative analysis of new bone were analysed to observe the new bone formation (Figure 5). More bone volume (BV) is visible in the BMP9-treated groups than in the GelMA/MS group (^*∗*^*p*  <  0.05) and BMSCs-GelMA/MS-GFP groups (^▲^*p*  <  0.05) at weeks 4 (Figure 5(a)). The BV of the BMSCs-GelMA/MS-BMP9 group is 19.82 mm^3^, representing 1553 times, 6.47 times, and 1.51 times higher than that of GelMA/MS, BMSCs-BMP9, BMSCs-GelMA/MS-GFP groups, respectively, at week 8 (Figure 5(b)). These results suggest that BMP9 and GelMA/MS could enable more bone formation. Furthermore, the number of trabeculae (Th.N) and trabeculae thickness (Tb.Th) show the same tendency of BV (Figures 5(c) and 5(d)). All the data indicate that the designed composite biomaterial (BMSCs-GelMA/MS-BMP9) enabled new bone formation by increasing the number and thickness of bone trabeculae *in vivo*.

**Figure 6:**
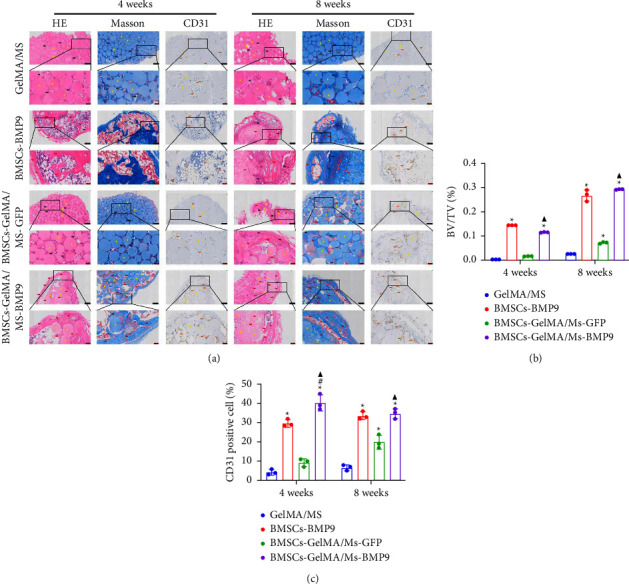
Histological staining and quantitative analysis (a) HE, Masson, and immunohistochemical staining of CD31 expression in the retrieved ectopic bone masses at 4 and 8 weeks. (b, c) quantitative analysis of bone volume fracture(BV/TV) in Masson staining and CD31^+^ cells in immunohistochemical staining (*n* = 3; ^*∗*^, ^#^, and ^▲^ indicate *p*  <  0.05 in comparison with the GelMA/MS group, the BMSCs-BMP9 group, and the BMSCs-GelMA/MS-GFP group, respectively; black and red bars = 200 *µ*m, 50 *µ*m).

HE, Masson, and immunohistochemical staining were performed to observe new bone (red arrow) and neovascularisation (brown arrow) (Figure 6(a)). CD31 is a marker of vascular endothelium [57], and the CD31^+^ cells are depicted as brown (brown arrow) by immunohistochemical staining, showing new blood vessels. A little bone formation with fibrous connective tissue (black arrow) around microspheres is observed in the GelMA/MS and BMSCs-GelMA/MS-GFP groups at weeks 4 and 8. Surprisingly, the amount of new bone formation, plenty of blood vessels, and irregularly arranged fibrous connective tissues are also found in the BMSCs-BMP9 group and BMSCs-GelMA/MS-BMP9 group at weeks 4 and 8. The new bone volume fractions (BV/TV) were investigated (Figure 6(b)), and it is significantly higher in the BMSCs-GelMA/MS-BMP9 group than BMSCs-GelMA/MS-GFP group (*p*  <  0.05), indicating that BMP9 enhanced more new bone. Furthermore, the BV/TV of the BMSCs-GelMA/MS-GFP group is also higher than the GelMA/MS group at week 8 (*p*  <  0.05), validating that BMSCs also improve new bone formation. The number of CD31^+^ cells in BMSCs-BMP9 and BMSCs-GelMA/MS-BMP9 groups are significantly higher than in the GelMA/MS and BMSCs-GelMA/MS-GFP groups (*p*  <  0.01) (Figure 6(c)), indicating that BMP9 promoted vascularisation. Meanwhile, the number of CD31^+^ cells in the BMSCs-GelMA/MS-BMP9 group are higher than in the BMSCs-BMP9 group at week 4, indicating that GelMA/MS promotes the formation of neovascularization. In combination, the novel compound implant (BMSCs-GelMA/MS-BMP9) achieved the best outcomes in forming new bone and degrees of neovascularisation *in vivo*, which BMP9 and GelMA/MS could enhance.

These results illustrate that BMP9 directly induces the osteogenic differentiation of BMSCs *in vitro* and neovascularisation *in vivo*, which is enhanced by GelMA/MS. The compound implant of Ad-BMP9 transfected BMSCs cocultured with GelMA presented the best osteogenic property outcomes both *in vitro* and *in vivo* and BMP9 directly induces the osteogenic differentiation of BMSCs *in vitro* and neovascularisation *in vivo.*

## 4. Discussion

Cells, growth factors, and scaffolds are the three critical elements of tissue engineering research [58–60]. This study demonstrates that BMSCs could thrive on the GelMA/MS scaffold produced from microfluidic devices, with BMP9 more vigorously promoting osteogenesis. Based on these findings, a novel implant comprising Ad-BMP9-transfected BMSCs and GelMA/MS was developed and constructed. This novel implant was injected *in vivo* beneath the skin of nude mice, and the results showed significant new vascularized bone formation.

As a multipotent cell, BMSCs derived from bone marrow tissue can differentiate into osteoblasts and have the potential for oral and maxillofacial bone regeneration [61–63]. This study illustrates that BMSCs treated with Ad-BMP9 had intense ALP activity and mineralised deposition. Furthermore, the RT-qPCR analysis of osteogenic marker genes, including *ALP*, *RunX2,* and *OPN,* was significantly induced by BMP9 *in vitro*, indicating that BMP9 could promote the osteogenic differentiation of BMSCs. This study observed more new bone tissue and blood vessels in the BMSCs-GelMA/MS-GFP group than in the GelMA/MS groups in mice with immunodeficiency. Applying BMSCs could be a valuable approach to promoting new bone regeneration, with BMP9 mediating the osteogenic activity of BMSCs and resident osteoblasts, fostering new bone formation.

GelMA is an ECM scaffold material that can be applied in tissue engineering for bone tissue regeneration. The diameter of GelMA/MS was approximately 150 *µ*m and could be injected through the 26G injector. This characteristic is advantageous for the minimally invasive treatment of bone defects. Many methods, such as batch emulsion, microfluidic, and photolithography, are available to prepare GelMA microspheres [64]. Microfluidic methods have superior advantages: (I) monodisperse microspheres in a specific size range can be obtained by regulating each channel's diameter size and flow rate. (II) The mild preparation environment is advantageous to the stability of GelMA physical chemistry and the material's biocompatibility. In this study, SEM and light microscopy illustrated that the microspheres had uniform morphology and reasonable swelling ratio, which could provide a humid environment for cells and increase the transportation of nutrients and oxygen diffusion for cell survival. The more a microsphere swells, the more it feeds the cells with a comfortable bioenvironment to live and function. The chemical properties of the microspheres show that GelMA/MS retained various chemical properties of GelMA, and the contact angle was reduced, which means that microfluidic did not change the physicochemical properties of the gel but increased hydrophilicity. The results of CCK-8, live/dead staining and phalloidin staining suggested that BMSCs could grow and expand on the surface of the microspheres, and GelMA/MS had excellent biocompatibility.

In this study, cells were selected to seed on microspheres instead of encased in the microspheres for the following reasons [65–67]: (I) the microspheres with a diameter of 100–400 *µ*m were suitable for encapsulating cells. In contrast, cells in microgels are restricted within the inner space of the gel, thereby limiting the ability of the cells to proliferate [67], (II) cells in the microsphere proliferate faster than those in the inner space of microspheres because the space is not tightly confined, (III) complex experimental conditions and procedures are required to wrap the cells in the microsphere. Compared to the “in the microsphere” strategy, the “on the microsphere” strategy generates microspheres before the cell growth, providing more flexibility in the choice of gelation conditions and precursor solutions, and (IV) UV light used during the manufacturing process of GelMA is harmful to cells and can reduce the survival rate of cells [68]. Regarding osteogenesis *in vivo*, a small amount of osteogenesis was evident in the simple GelMA/MS scaffold material, inferring that GelMA/MS could play a stable role in osteogenesis, promoting osteoblasts to secrete osteogenic factors between scaffolds to form bone. Compared with the BMSCs-BMP9 group, the scaffold-containing BMSCs-GelMA/MS-BMP9 experiment yielded a higher total volume of bone formation, suggesting that GelMA/MS acts as a scaffold in new bone formation and encourages bone regeneration.

Ad-BMP9-transfected BMSCs combined with GelMA/MS enhance new bone formation. BMP9 is among the most osteogenic BMPs, promoting osteoblastic differentiation compared to other BMP family members [69–71]. Recently, some successful *in vitro* and *in vivo* research suggests that BMP9 might be a more effective strategy for promoting bone regeneration. Delivery of the gene via adenoviral vectors can be better controlled and more efficient than delivery of the protein [37]. The osteogenic activity induced by recombinant human bone morphogenetic protein 9(rhBMP9) was significantly weaker than Ad-BMP9 [38]. The ALP activity and staining results, alizarin red S staining, and RT-qPCR revealed that the combination of Ad-BMP9-transfected BMSCs and GelMA/MS could significantly enhance the osteogenic differentiation of BMSCs and osteogenic activity. BMP9 mediated the osteogenic activity of BMSCs and resident osteoblasts.

Furthermore, this study also determined whether the new combination therapy of Ad-BMP9-transfected BMSCs and GelMA/MS was possible for oral and maxillofacial bone tissue engineering and future clinical use. The immunohistochemical staining revealed that more blood vessels were found in BMP9-treated groups than those without BMP9, indicating that BMP9 promoted angiogenesis. A previous study showed sinusoid capillary formation is observed early in BMP9-induced bone formation [35]. Meanwhile, the BMSCs-GelMA/MS-BMP9 group were higher than the BMSCs-BMP9 group at week 4, indicating that GelMA/MS also promoted the formation of neovascularization. Angiogenesis is very important for successful bone regeneration [72]. Blood vessels are not only a source of oxygen and nutrients but also provide calcium and phosphate, the building blocks of mineralization [72]. The micro-CT data, HE and Masson staining verified this result and depicted more new bone in the BMSCs-GelMA/MS-BMP9 group than in other groups. BMP9-induced bone formation is coupled with angiogenesis, which promotes both osteogenesis and angiogenesis. The more neovascular and new bone tissue levels were detected in the BMP9-treated groups compared with other groups without BMP9. Alternately, the three-dimensional environment constructed by the microspheres is conducive to the growth and proliferation of cells [73]. Studies [73, 74] have shown that stem cells cultured in three-dimensional environment had higher stemness, showed better differentiation potential and secreted more associated transcription factors than those cultured in a two-dimensional environment. Notably, BMP9 can promote both blood vessel formation and osteogenesis. BMP9 is also synergistic for hematopoietic progenitor cell generation [75]. BMP9 is one of the most osteogenic BMPs, promoting osteoblast differentiation of mesenchymal stem cells *in vitro* and *in vivo* [76]. BMP9 may induce convergence of osteogenic and angiogenic signals during MSC differentiation, improving the efficiency of bone formation and development [77].

The novel compound implants of Ad-BMP9-transfected BMSCs and GelMA/MS exerted the best effect on new bone formation beneath the skin of immunodeficient mice. However, there are many challenges, such as the security of this new bioimplant, the long-term effectiveness, and the evaluation of this method, before realising the full clinical potential of this combination.

## 5. Conclusion

In conclusion, this study designed and assembled a new type of implant with Ad-BMP9-transfected BMSCs, and GelMA/MS made from microfluidics. GelMA/MS has good biocompatibility and hydrophilicity, and the BMP9 boosted the BMSCs' osteogenic differentiation on the biodegradable GelMA/MS scaffold. The tissue-engineered technique combined with Ad-BMP9-transfected BMSCs and GelMA/MS achieved the best effects in new vascularized bone formation with this novel implant. These findings might lead to a novel approach for treating bone defects in oral and maxillofacial regions.

## Data Availability

The data used to support the findings of this study are included within the article and supplementary information files.

## Supplementary Materials

Supplementary Materials Graphical abstract GelMA microspheres (GelMA/MS) were made by two microfluidic synchronous cross-linking device and could be stored for a long time after lyophilization. BMSCs, transfected with Ad-BMP9, were cocultured on GelMA/MS for 3 days. Then, the bioimplants composed of Ad-BMP9-transfected BMSCs and GelMA/MS were injected beneath the skin of nude mouse by a 26G injector for new bone formation. Figure S1: A and B: TGA analysis; B: porosity; C: zeta potential. Figure S2: characterization of GelMA/MS and GelMA (A) water contact angle; (B) XRD; (C) XPS; (D) FTIR; (E) NMR. Figure S3: real-Time qPCR quantitative analysis of osteogenic genes, including alkaline phosphatase (ALP), osteopenia (OPN) and runt-related transcription factor 2 (RunX2) (*n* = 3, ^*∗*^, ^▲^ and ^#^ indicate *p*  <  0.05 in comparison with GFP group, GelMA/MS-GFP group and BMP9 group respectively. ^*∗∗*^ and ^##^ indicate *p*  <  0.01 in comparison with GFP group and GelMA/MS-GFP group respectively). Figure S4: the images of new bone tissue at week 4 and 8.
