# Supplementary material for: A Novel Bioimplant Comprising Ad-BMP9-Transfected BMSCs and GelMA Microspheres Produced from Microfluidic Devices for Bone Tissue Engineering
Source: J Tissue Eng Regen Med. 2023 Jun 19;2023:2981936. doi: 10.1155/2023/2981936 (PMC11918572; doi:10.1155/2023/2981936)
Supplement: Supplementary Materials — Graphical abstract GelMA microspheres (GelMA/MS) were made by two microfluidic synchronous cross-linking device and could be stored for a long time after lyophilization. BMSCs, transfected with Ad-BMP9, were cocultured on GelMA/MS for 3 days. Then, the bioimplants composed of Ad-BMP9-transfected BMSCs and GelMA/MS were injected beneath the skin of nude mouse by a 26G injector for new bone formation. Figure S1: A and B: TGA analysis; B: porosity; C: zeta potential. Figure S2: characterization of GelMA/MS and GelMA (A) water contact angle; (B) XRD; (C) XPS; (D) FTIR; (E) NMR. Figure S3: real-Time qPCR quantitative analysis of osteogenic genes, including alkaline phosphatase (ALP), osteopenia (OPN) and runt-related transcription factor 2 (RunX2) (n = 3, ∗, ▲ and # indicate p < 0.05 in comparison with GFP group, GelMA/MS-GFP group and BMP9 group respectively. ∗∗ and ## indicate p < 0.01 in comparison with GFP group and GelMA/MS-GFP group respectively). Figure S4: the images of new bone tissue at week 4 and 8. [file 2981936.f1.docx]

**A Novel Bio-Implant Comprising Ad-BMP9-Transfected BMSCs and GelMA Microspheres Produced from Microfluidic Devices for Bone Tissue Engineering**

Li Nie^1, 2, 3^, Wei Liu^1, 2, 3^, Jiajun Chen^1, 2, 3^, Siqi Zhou^1, 2, 3^, Chang Liu^1, 2, 3^, Wenhui Li^1, 2, 3^, Zhiyue Ran^1, 2, 3^, Yaxian Liu^1, 2, 3^, Jing Hu^1, 2, 3^, Yuxin Zhang^1, 2, 3^, Liwen Zheng^1, 2, 3^, Ping Ji^1, 2, 3^ †, Hongmei Zhang^1, 2, 3^ †

^1^ Stomatological Hospital of Chongqing Medical University, Chongqing, 401147, China.

^2^ Chongqing Key Laboratory of Oral Diseases and Biomedical Sciences, Chongqing Municipal Key, Chongqing, 401147, China.

^3^ Laboratory of Oral Biomedical Engineering of Higher Education, Chongqing, 401147, China.

† Co-corresponding author: Hongmei Zhang(hmzhang@hospital.cqmu.edu.cn), Ping Ji ([jiping@hospital.cqmu.edu.cn](mailto:jiping@hospital.cqmu.edu.cn))

**Key word:** Bone morphogenetic protein 9, BMSCs, bone tissue engineering, GelMA, microfluidics.

 Supplementary Figures


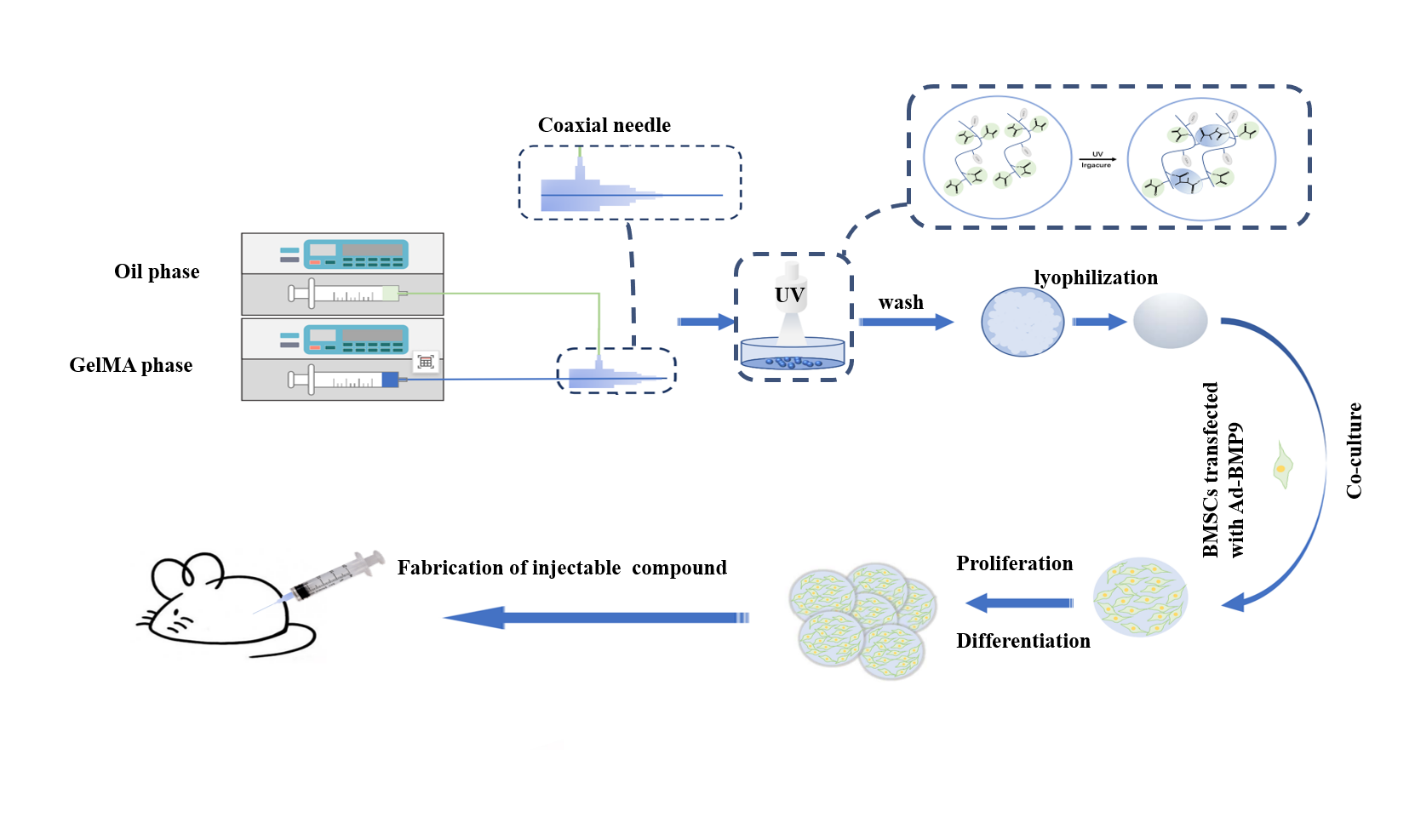


**Graphical abstract** GelMA microspheres (GelMA/MS) were made by two microfluidic synchronous cross-linking device and could be stored for a long time after lyophilization. BMSCs, transfected with Ad-BMP9, were co-cultured on GelMA/MS for 3 days. Then the bio-implants composed of Ad-BMP9-transfected BMSCs and GelMA/MS were injected beneath the skin of nude mouse by a 26G injector for new bone formation.


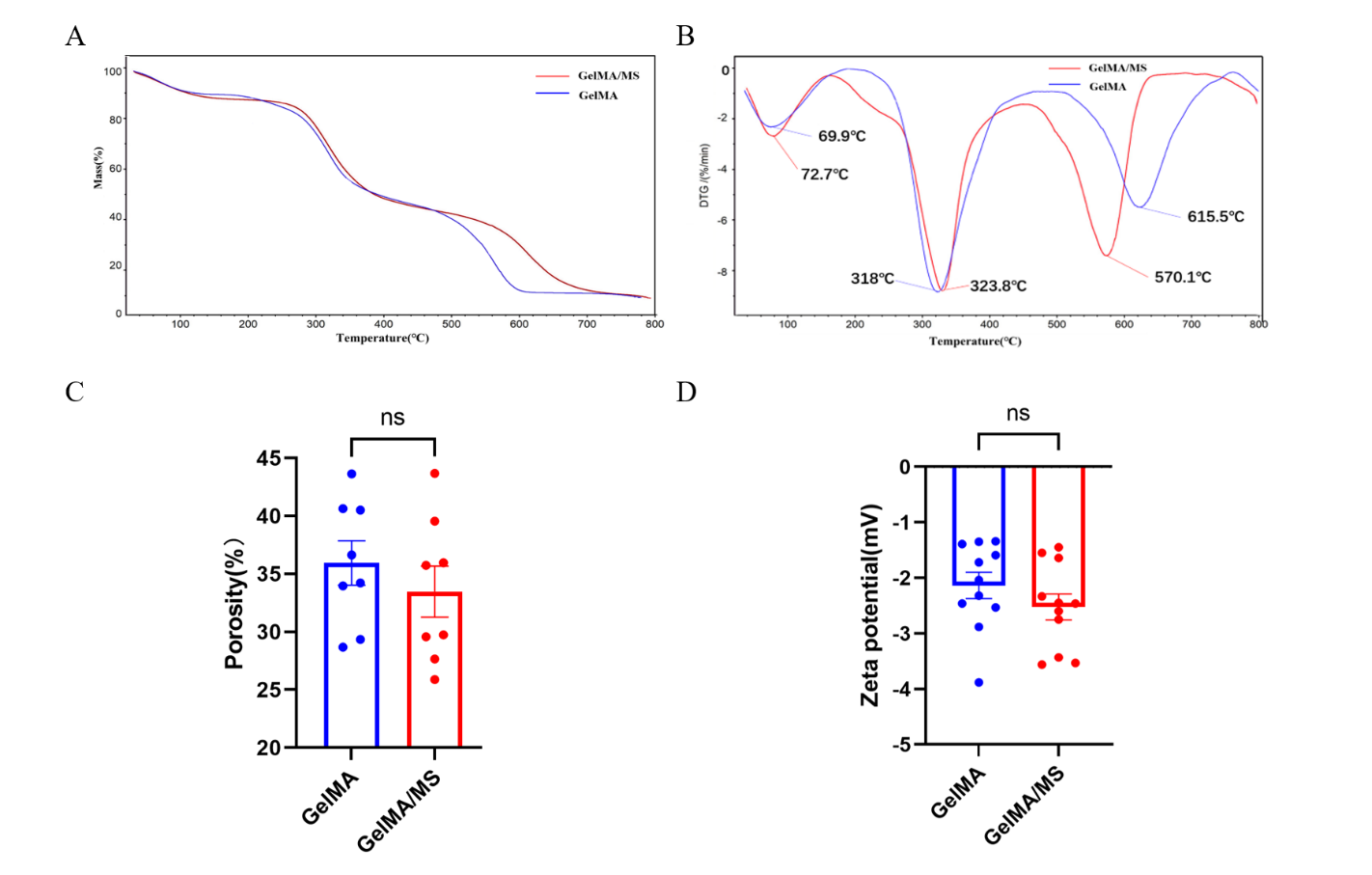


**Fig. S1** A and B: TGA analysis; B: porosity; C: zeta potential


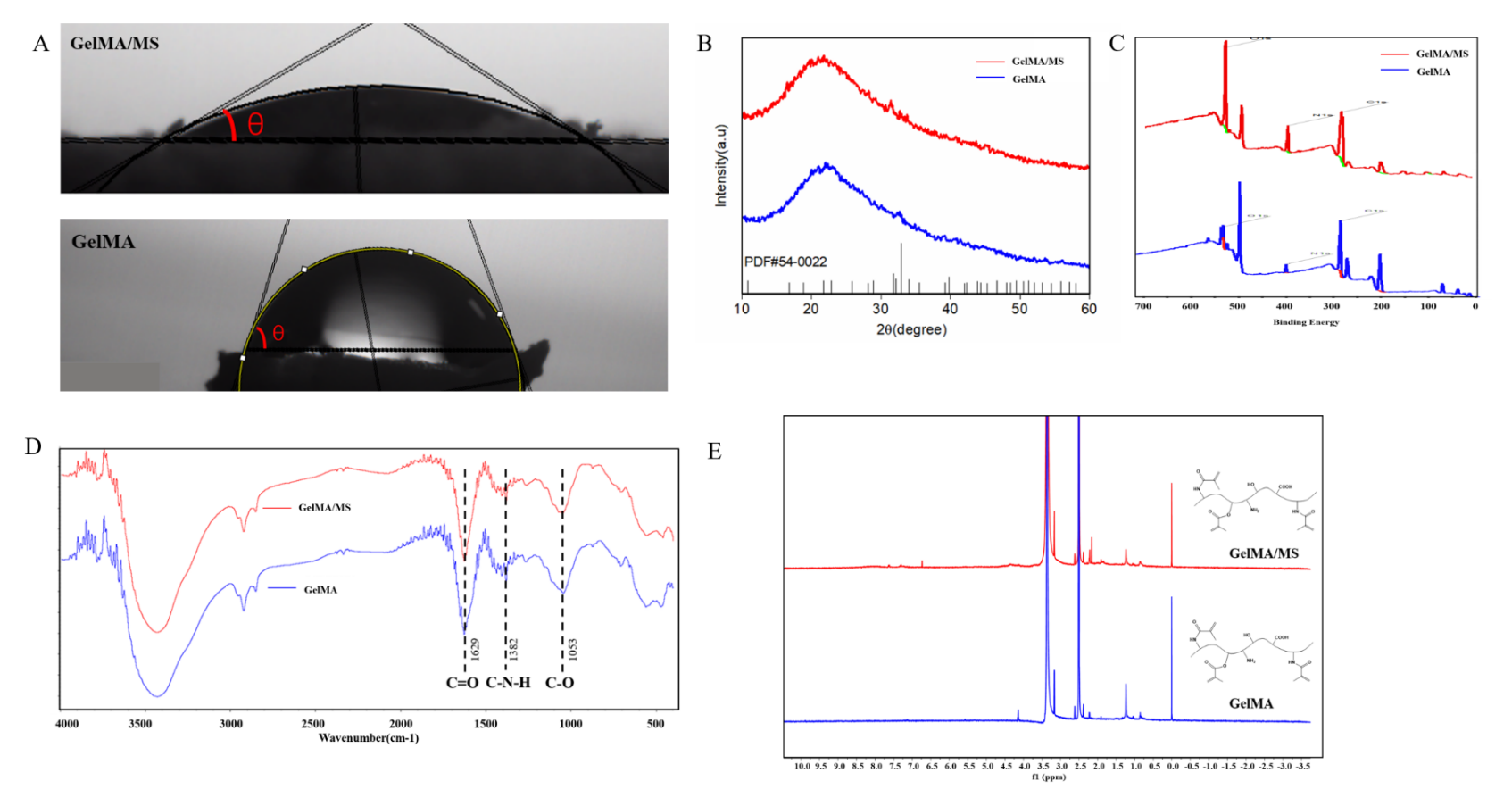


**Fig. S2** Characterization of GelMA/MS and GelMA A: water contact angle; B: XRD; C: XPS; D: FTIR; E: NMR.


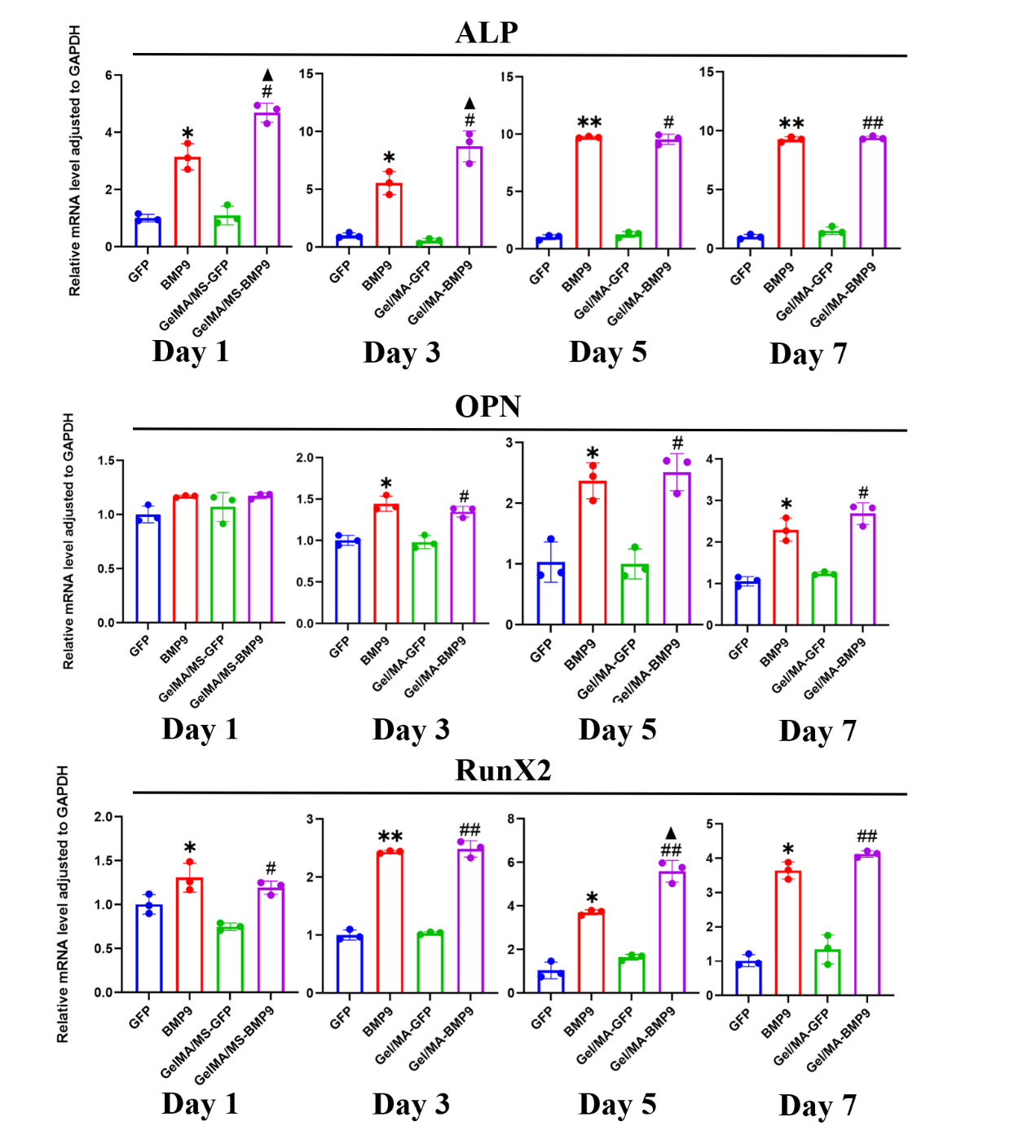


**Fig. S3** Real Time-qPCR quantitative analysis of osteogenic genes, including alkaline phosphatase (*ALP*), Osteopenia (*OPN*) and runt-related transcription factor 2 (*RunX2*) (n = 3, *, ▲ and # indicate *p* < 0.05 in comparison with GFP group, GelMA/MS-GFP group and BMP9 group respectively. **and ## indicate *p* < 0.01 in comparison with GFP group and GelMA/MS-GFP group respectively).


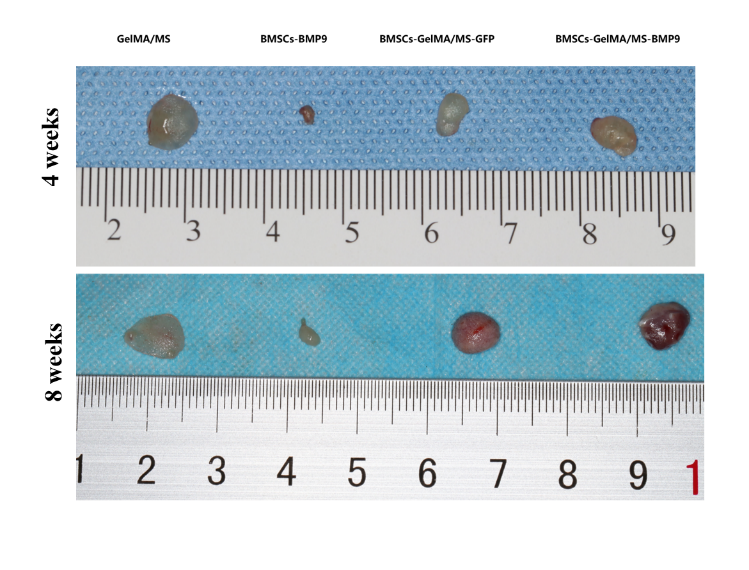


**Fig. S4** The images of new bone tissue at week 4 and 8.
